# Whole-Exome Sequencing Uncovers Specific Genetic Variation Difference Based on Different Modes of Drug Resistance in Small Cell Lung Cancer

**DOI:** 10.3389/fonc.2022.891938

**Published:** 2022-06-30

**Authors:** Ning Tang, Zhenzhen Li, Xiao Han, Chenglong Zhao, Jun Guo, Haiyong Wang

**Affiliations:** ^1^ Department of Internal Medicine-Oncology, Shandong Cancer Hospital and Institute, Shandong First Medical University and Shandong Academy of Medical Sciences, Jinan, China; ^2^ Berry Oncology Corporation, Beijing, China; ^3^ Department of Pathology, The First Affiliated Hospital of Shandong First Medical University and Shandong Provincial Qianfoshan Hospital, Jinan, China

**Keywords:** small cell lung cancer, whole-exome sequencing, somatic mutational signature, recurrence, therapeutic strategy

## Abstract

The poor survival rate of small cell lung cancer (SCLC) is mainly related to the condition that patients with SCLC often have good responses to first-line chemotherapy initially, but later on, most of these patients relapse rapidly due to resistance to further treatment. In this study, we attempted to analyze whole-exome sequencing data based on the largest sample size to date, to develop a classifier to predict whether a patient will be chemorefractory or chemosensitive and to explicate the risk of recurrence that affects the prognosis of patients. We showed the different characteristics of somatic mutational signatures, somatic mutation genes, and distinct genome instability between chemorefractory and chemosensitive SCLC patients. Amplified mutations in the chemosensitive group inhibited the regulation of the cell cycle process, transcription factor binding, and B-cell differentiation. Analysis of deletion mutation also suggested that detection of the chromosomal-level variation might influence our treatment decisions. Higher PD-L1 expressions (based on TPS methods) were mostly present among chemosensitive patients (*p* = 0.026), while there were no differences in PD-L1 expressions (based on CPS methods) and CD8^+^ TILs between the two groups. According to the model determined by logistic regression, each sample was endowed with a predictive probability value (PV). The samples were divided into a high-risk group (>0.55) and a low-risk group (≤0.55), and the survival analysis showed obvious differences between the two groups. This study provides a reference basis to translate this knowledge into practice, such as formulating personalized treatment plans, which may benefit Chinese patients with SCLC.

## Introduction

Lung cancer remains one of the most frequently diagnosed cancers and the most common cause of cancer-related mortality worldwide, with small cell lung cancer (SCLC) accounting for ~15% of all lung cancer cases (1, 2). SCLC is characterized by rapid growth, a tendency to metastasize, and poor survival rates with a median survival rate of 7 months. Patients with SCLC often have good responses to first-line chemotherapy initially; however, most of these patients relapse rapidly due to resistance to further treatment. Therefore, SCLC has been classified as a chemorefractory disease if patients develop resistance to platinum-based chemotherapy within 3 months. In a situation where the disease has been controlled for 3 months or longer, it is defined as chemosensitive (3). Thus, there is an urgent need to predict whether a patient is chemorefractory or chemosensitive and to explicate the risk of recurrence that may affect the prognosis of patients.

Recently, researchers have attempted to use single-cell sequencing of circulating tumor cells (CTCs) to develop classifiers by bioinformatics analysis of the genome-wide copy number alteration (CNA) data. Carter and colleagues (4) reported that they identified 2,281 loci from blood samples drawn from 13 patients with SCLC that indicated substantial discrepancy to generate 16 CNA profiles that stratified CTC samples into chemosensitive and chemorefractory patients. Su et al. (5) established a 10-CNA score classifier based on single CTCs from 48 patients for the prediction of prognosis, which demonstrated that a high CNA score could herald poor PFS. We have observed different results among these studies, which warrants a larger cohort. In addition, the stability of liquid biopsies could be influenced by tumor location, size, vascularity, and the detection method used (6, 7); thus, the results based on tumor tissues are expected to be seen. Moreover, immune checkpoint inhibitors (ICIs) have become the paradigm for the treatment of cancer (3, 8–11). The first-line management of extensive-stage SCLC has been platinum with etoposide; however, the addition of atezolizumab or durvalumab to chemotherapy resulted in superior overall survival compared with platinum and etoposide treatment. The influence of the tumor immune microenvironment on resistance in patients with SCLC is generally less studied.

In this study, we attempted to analyze the whole-exome sequencing (WES) data based on the tissues of 177 SCLC patients, known to be the largest sample size currently, to develop a classifier covering the clinical features, tumor immune microenvironment, gene mutation, and chromosome structure variation, in the hope of improving the precise and appropriate treatment for patients with SCLC. We hope that these endeavors would lead to the precise treatment of SCLC patients.

## Material and Methods

### Sample Collection, Processing, and Genomic DNA Extraction

We recruited histologically confirmed SCLC patients from the Shandong Cancer Hospital and Institute (SCH). All diagnoses were independently confirmed by two experienced pathologists. In addition to blood samples (2 ml), tumor tissue samples were obtained by biopsies. A strict quality inspection was carried out to remove contaminated and insufficient DNA samples. Finally, 177 patients were enrolled in our study. The overall survival (OS) time was defined as the interval between diagnosis and death, or between diagnosis and the last observation point. For surviving patients, data were censored at the last follow-up (November 26, 2020). Clinicopathological data were retrieved from the patients’ medical records. This study was approved by the Ethics Committee of the Shandong Cancer Hospital and Institute. All patients included in this study provided written informed consent.

Biopsied tumor tissues were fixed in formalin and then embedded in paraffin (FFPE). The corresponding blood samples were set as controls. Genomic DNA was extracted from each FFPE sample using the GeneRead DNA FFPE Kit (Qiagen, USA) and from the blood sample using the DNA Blood Midi/Mini Kit (Qiagen, USA).

### DNA Library Construction and Whole-Exome Sequencing

Genomic DNA was enzymatically digested into 200 bp fragments (5× WGS Fragmentation Mix, Qiagen, USA). T-adapters were added to both ends after repairing and A tailing. For the WES library construction, purified DNA was amplified by ligation-mediated PCR. Then, final sequencing libraries were generated using the 96 rxn xGen Exome Research Panel v1.0 (Integrated DNA Technologies, USA), according to the manufacturer’s instructions. Paired-end multiplex samples were sequenced with the Illumina NovaSeq 6000 System (Illumina, USA). The sequencing depth was 200× per tissue sample and 100× per white blood cell (WBC) sample.

### Sequence Data Processing and Alignment of the SCH Cohort

Raw sequencing data were preprocessed by Fastp to trim adaptor sequences (12). Then, clean reads in FastQ format were aligned to the reference human genome (hg19/GRCh37) by Burrows-Wheeler Aligner (BWA, v0.7.15) (13). SAM tools (14) and Picard (2.12.1) (http://picard.sourceforge.net/) were used to sort mapped BAM files and process PCR duplicates. To compute the sequencing coverage and depth, the final BAM files were generated by GATK (Genome Analysis Toolkit 3.8) for local realignment and base quality recalibration (15).

### Mutational Signature Analysis

Somatic mutational signatures were *de-novo* analyzed from the clean WES data by the “Somatic Signatures” R package (v2.20.0) (16), according to the non-negative matrix factorization (NMF) method. Four highly confident mutational signatures were derived from the SCH cohort. Then, they were compared with the consensus signatures in the COSMIC dataset (https://cancer.sanger.ac.uk/cosmic/), based on the cosine similarity analysis to nominate each derived signature with the highest COSMIC dataset similarity [i.e., SBS4 (S4), SBS2 (S2), SBS6 (S6), and SBS5 (S5), respectively] for the SCH cohort.

To further determine the distribution of mutational signatures and the frequencies of each patient, deconstructSigs (v1.9.0) was used as previously described (17). Patients harboring the S2, S4, S5, and S6 mutations, as well as S2, S4, S5, and S6 weights, were compared using the Wilcoxon test among the two groups.

### Somatic Mutation Variant Detection and Driver Gene Prediction

Somatic single nucleotide variations (SNVs) were identified from the clean sequencing data by MuTect (18), and somatic small insertions and deletions (InDels) were detected by the GATK Somatic Indel Detector. The ANNOVAR software was used for the annotation of variants based on multiple databases (19), including variant (HGVS), population frequency (1000 Genomes Project, dbSNP, ExAC), variant functional prediction (PolyPhen-2 and SIFT), and phenotype or disease (OMIM, COSMIC, ClinVar) databases. After the annotation, the retained non-synonymous SNVs were screened from disease databases for further analysis with variant allele frequency (VAF) (cutoff ≥ 3%) or VAF for cancer hotspots (cutoff ≥ 1%). Tumor mutation burden (TMB) was calculated with the total numbers of non-synonymous SNVs and indel variants per megabase of coding regions. Dominant tumor neoantigens were predicted using OptiType to infer the individual HLA type (20). Tumor neoantigen burden (TNB) was calculated with the total numbers of neoantigens per megabase of coding regions. Significant driver genes were identified by combining MutsigCV and dNdScv, as previously described (21, 22), with a false discovery rate (FDR) cutoff <5%. Genes with significantly different mutation frequencies among the two groups were determined based on the gene mutation rates in each group using a two-sided Fisher’s exact test with a *p*-value of 0.05.

### Copy Number Variation Identification

Copy number variations (CNVs) for all patients in the SCH cohort were first identified using the Genome Identification of Significant Targets in Cancer (GISTIC) 2.0 algorithm (23). At the chromosomal arm level, significant amplifications or deletions were screened with FDR (cutoff < 10%) for further analyses. At a focal CNV level, significant amplification was screened with FDR (cutoff < 5%) and G-score (cutoff > 0.3). Significant deletion was screened with FDR (cutoff < 5%) and G-score (cutoff < −0.2) for further analyses.

Focal CNV-related gene analysis was performed for each patient based on paired tumor-normal WES data using the GATK depth of coverage with parameters (–minBaseQuality 0 –minMappingQuality 20 –start 1 –stop 500 –nBins 200 –includeRefNSites –countType COUNT_FRAGMENTS). The amplified genes were defined by a copy number ratio of tumor vs. normal >4, while deleted genes were defined by a copy number ratio of tumor vs. normal <0.5. Then, focal CNV-related genes were filtered according to the COSMIC Cancer Gene Census database (https://cancer.sanger.ac.uk/cosmic/) to obtain a cancer-related focal CNV gene list. Genes with significantly different CNV frequencies among the two groups were determined based on the gene alteration rates in each group using a two-sided Fisher’s exact test with a *p*-value of 0.05.

### Pathway and Functional Enrichment Analysis

Somatic mutation and focal CNV-related genes with enriched biological functions and involved pathways were analyzed using the online tool Metascape (https://metascape.org/gp/index.html#/main/step1), based on the Gene Ontology (GO) and Kyoto Encyclopedia of Genes and Genomes (KEGG) databases (https://www.kegg.jp/kegg/kegg1.html).

### Tumor Heterogeneity and Genome Instability Analysis

To investigate intratumor heterogeneity (ITH), mutant allele tumor heterogeneity (MATH) values for each tumor sample were calculated from the median absolute deviation (MAD) and the median of its mutant–allele fractions at tumor-specific mutated loci: MATH = 100 × MAD/median. These analyses were performed in R with default parameters as previously reported (24). Cancer cell fraction (CCF) and clonal and subclonal mutations in each tumor specimen were calculated based on the proportion of mutated reads (VAF) as previously reported (25).

Regarding genome instability analyses, cellular purity, ploidy, and the segmented allele-specific copy number profiles of each specimen’s tumor cells were estimated using Sequenza (26). The fraction of genome altered (FGA) was defined as the percentage of a tumor genome harboring copy number variations against the whole genome. Loss-of-heterozygosity (LOH) segments or mutations were defined by the minor allele copy number or mutation ratio <0.25 (27). Whole-genome doubling (WGD) events were defined as the major allele ploidy >1.5 on at least 70% of at least 11 autosomes as the duplicated autosome number per sample (28). The Wilcoxon rank-sum test was used to compare the median values of the variables between the two groups. A *p*-value of 0.05 was considered significant.

### Immunohistochemical Staining

Immunohistochemical staining was conducted using the Enhance Labelled Polymer System (ELPS). First, the tumor specimen sections were incubated with anti-PD-L1 (CST, 13684, 1:100) and anti-CD8^+^ (CST, 85336, 1:100) at 4°C overnight. Then, they were washed three times with PBS (5 min per wash). Next, the slides were incubated with the corresponding secondary antibodies at 37°C for 30 min, and they were washed three times with PBS (5 min per wash). Furthermore, the slides reacted with 3,3-diaminobenzidine (DAB) and then washed with distilled water. Next, dehydration was conducted, followed by clearing and mounting with neutral gums. Finally, the stained tissue images were captured by the Digital Pathology Slide Scanner (KF-PRO-120, KF-BIO).

### Programmed Cell Death Ligand 1 Expression

To evaluate programmed cell death ligand 1 (PD-L1) expression, a tumor proportion score (TPS) was defined as the number of PD-L1-staining tumor cells divided by the total number of viable tumor cells multiplied by 100. A combined positive score (CPS) was defined as the number of PD-L1-staining cells divided by the total number of viable tumor cells multiplied by 100 (29). Tonsil PD-L1 staining was adopted to ensure the eligibility of the enrolled specimens. Qualified staining was defined as strong positivity for PD-L1 in the intratonsillar cleft epithelium, whereas negative staining was for PD-L1 in lymphocytes (mantle zone and germinal center B cells) and superficial epithelial cells.

### CD8^+^ T-Cell Infiltration

We also evaluated whether CD8^+^ T cells were uniformly distributed in the tumor stroma at lower magnification. If CD8^+^ T cells were equally distributed, they were measured in three randomly chosen areas (0.1 mm^2^) at a 200-fold magnification. If unequally distributed, the corresponding areas were selected at a 200-fold magnification according to CD8^+^ T-cell percentages in areas of different densities (0.1 mm^2^), as referred from the PD-L1 expression evaluation criteria. The calculation was defined as follows: CD8^+^ T-cell count/0.1 mm^2^ × 10 or CD8^+^ T-cell count/mm^2^.

### Sequencing Data Availability

Raw sequencing data were deposited in the Genome Sequencing Archive (GSA) of the China National Center for Bioinformation (CNCB) (https://ngdc.cncb.ac.cn/gsa/) under accession number subHRA001430.

### Statistical Analyses

The R Foundation for Statistics Computing Package (R package, version 3.3.3) was used to perform the statistical analyses. The Fisher’s exact test (for categorical variables) and the Wilcoxon rank-sum test (for continuous variables) were used to analyze the relationship between the two groups. The Kaplan–Meier method was used to estimate the effects on OS and PFS time based on the log-rank tests. A *p*-value <0.05 was defined as statistically significant. Hazard ratios of multiple factors on OS and PFS time were obtained from the Cox proportional hazards model.

## Results

### Different Characteristics of Somatic Mutational Signatures Between Chemorefractory and Chemosensitive SCLC Patients

A further expedition of SCLC genomic landscape features was presented through the WES of the large SCH SCLC cohort. First, the mutation spectrum analysis showed that the two most frequent nucleic acid base substitutions of somatic mutations were transversions (C>A/G>T) followed by transitions (C>T/G>A) (**Supplementary Figure 2**), consistent with previous SCLC studies. Then, mutational signatures were *de-novo* calculated and characterized from all 177 specimens based on the 96 possible mutation types, according to a previously published method.

**Figure 1:**
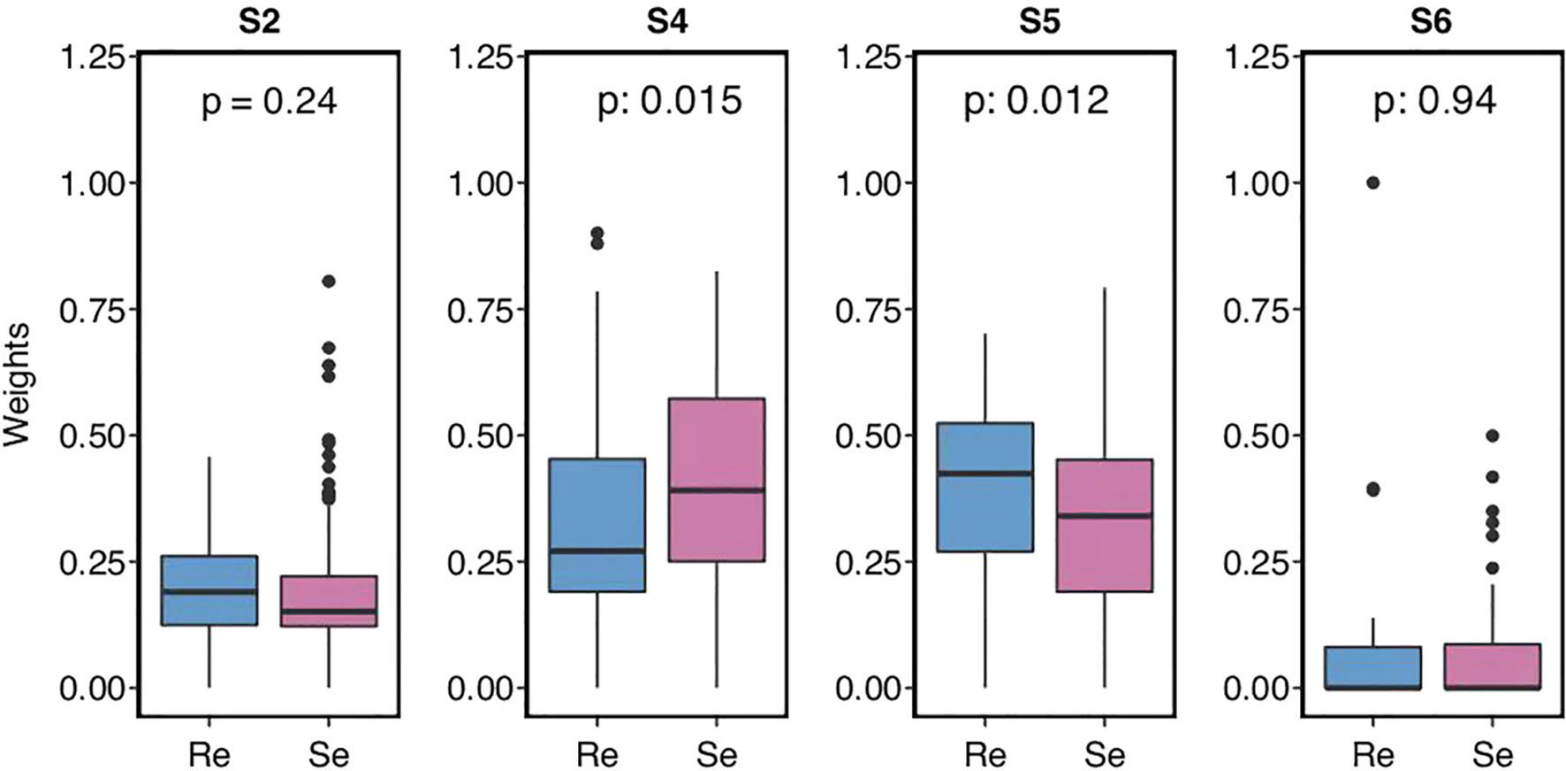
Small cell lung cancer (SCLC) patients’ mutational signature and the weights of different somatic mutational signatures in each group.

Two signatures showed differences between the two groups, compared to the COSMIC mutational signature database: smoking-related S4 and unknown S5 (**Figure 1**). Generally, SCLC is associated with heavy tobacco smoking. Chemosensitive patients tended to harbor smoking-related S4 mutations more than chemorefractory patients. However, smoking history did not significantly differ between the two groups. The situation had previously been reported that C>A/G>T transversions, typically prevalent in S4, have no significant correlation with the SCLC smoking status (30, 31). In turn, S5 mutations were more frequently found in the chemorefractory group. This indicated that therapeutic vulnerabilities for different SCLC subtypes may be related to molecular change during SCLC tumor development.

### Driver and Recurrent Somatic Mutation Genes Between the Two Groups

**Figure 2:**
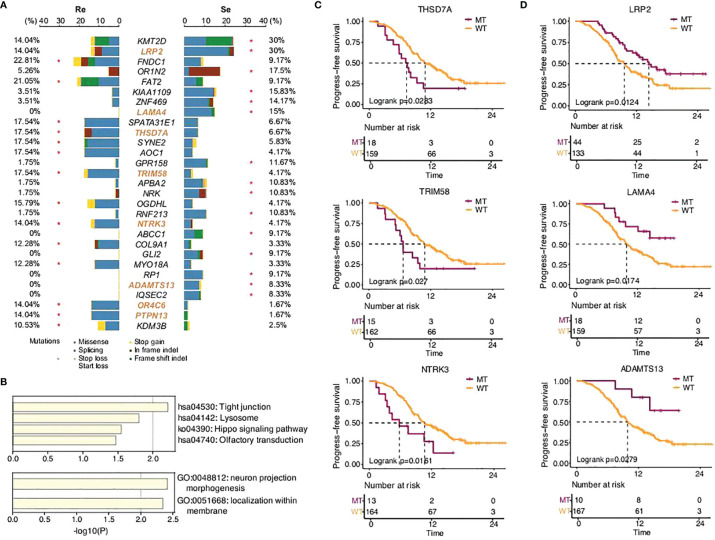
SCLC patients’ somatic mutational features in the two groups. **(A)** Comparison of somatic mutational features with a mutation frequency greater than 5%. **(B)** GO functions enriched by all the genes predicted in this study. KEGG pathways enriched by the somatic mutation genes that significantly affected PFS time in this study. **(C,D)** Progression-free survival of different gene status.

SCLC is considered to be a disease of genomic alterations and relatively higher mutation rate per megabase (Mb) (1, 2). We used MutSigCV and dNdScv (FDR *q* < 0.1) to identify the differential somatic mutation genes between the two groups, and the genes with a mutation frequency greater than 5% were indicated in the graph (**Figure 2A**). Our results showed that *KMT2D* (30%), *LRP2* (30%), *OR1N2* (17.5%), *KIAA1109* (15.83%), *LAMA4* (15%), *ZNF469* (14.17%), *GPR158* (11.67%), *NRK* (10.83%), *APBA2* (10.83%), *RNF213* (10.83%), *ABCC1* (9.17%), *GLI2* (9.17%), *RP1* (9.17%), *ADAMTS13* (8.33%), and *IQSEC2* (8.33%) were more frequently predicted in the chemosensitive group, while *FNDC1* (22.81%), *FAT2* (21.05%), *SPATA31E1* (17.54%), *AOC1* (17.54%), *SYNE2* (17.54%), *THSD7A* (17.54%), *TRIM58* (17.54%), *OGDHL* (15.79%), *NTRK3* (14.04%), *OR4C6* (14.04%), *PTPN13* (14.04%), *COL9A1* (12.28%), *MYO18A* (12.28%), and *KDM3B* (10.53%) more commonly appeared in the chemorefractory group. This suggested the relativity between the high-frequency mutations of somatic mutation genes and disease recurrence.

Further functional analysis showed that the differential genes more frequently predicted in the chemorefractory group were most significantly enriched in tight junction, lysosome, Hippo signaling pathway, and olfactory transduction. Moreover, the differential genes more frequently predicted in the chemosensitive group were most significantly enriched in neuron projection morphogenesis and localization within the membrane (**Figure 2B**). The results suggested the potential mechanisms of chemoresistance on somatic mutation levels, which warrants further study.

In addition, among the differential genes found between the chemorefractory group and the chemosensitive group, we applied survival correlation analysis and identified eight somatic mutation genes mentioned earlier that significantly reduced PFS time, compared to wild-type genotypes (**Figure 2C** **;** **Supplementary Figures 3**, **4**). Meanwhile, three somatic mutation genes that were more frequently predicted in the chemosensitive group significantly increased PFS time. This further strengthens the (**Figure 2D**) fact that they have pivotal roles in SCLC relapse and chemotherapy resistance. There was no significant difference, however, in OS time apart from *LRP2* (**Supplementary Figure 5**).

The VAF analysis reflected that VAF in the Re group was higher than that in the Se group in all gene mutations (*p* = 6.2e−11) and clonal gene mutations (*p* = 0.022), but there was no difference concerning driver mutations (*p* = 0.41) or LOH (*p* = 0.27). No difference was observed between the two groups with respect to TMB (*p* = 0.12) and MATH score (*p* = 0.28) (**Supplementary Figure 6**).

### Distinct Genome Instability Between the Two Groups

**Figure 3:**
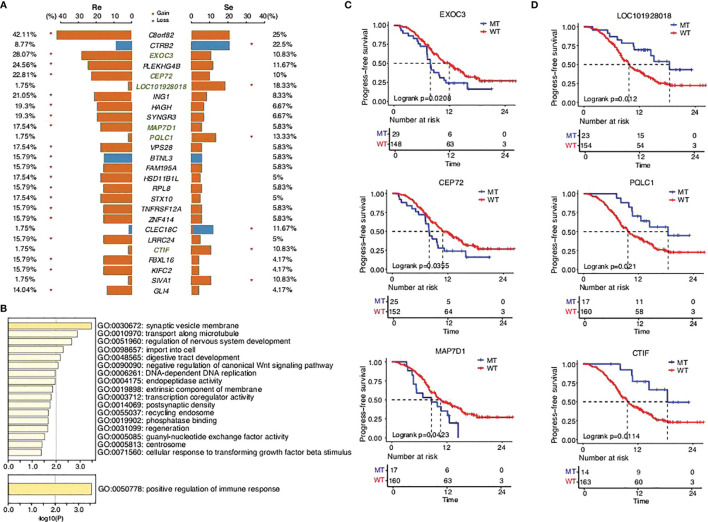
SCLC patients’ copy number variant in the two groups. **(A)** Comparison of the copy number variants with a mutation frequency greater than 5%. **(B)** GO functions enriched by all the mutations predicted in this study. KEGG pathways enriched by the mutations that significantly affected PFS time in this study. **(C,D)** Progression-free survival status of the different genes.

CNVs, novel structural variations in human chromosomes, are extremely common in SCLC, and our results had come to similar conclusions (**Figure 3**). We identified a high frequency of significant mutations in the two groups: *C8orf82CEP72* (42.11%), *EXOC3* (28.07%), *PLEKHG4B* (24.56%), *CEP72* (22.81%), *ING1* (21.05%), *SYNGR3* (19.3%), *HAGH* (19.3%), *MAP7D1* (17.54%), *VPS28* (17.54%), *STX10* (17.54%), *HSD11B1L* (17.54%), *FAM195A* (15.79%), *BTNL3* (15.79%), *RPL8* (15.79%), *ZNF414* (15.79%), *TNFRSF12A* (15.79%), *LRRC24* (15.79%), *KIFC2* (15.79%), *FBXL16* (15.79%), and *GLI4* (14.04%) in the chemorefractory group and *CTRB2* (22.5%), *LOC101928018* (18.33%), *PQLC1* (13.33%), *CLEC18C* (11.67%), *CTIF* (10.83%), and *SIVA1* (10.83%) in the chemosensitive group. The above somatic CNV analyses showed that the chemorefractory group seemingly experienced more mutations of the CNV site compared with the chemosensitive group (**Figure 3A**). We have noted that these changes involved certain oncogenic signaling pathways (**Figure 3B**) whose alterations significantly impacted patients’ PFS time (**Figure 3C** **;** **Supplementary Figures 7**-**11**). Similar to previous results, these changes had very little impact on OS time (**Supplementary Figures 7**-**12**).

### Amplified or Deletion Mutations Between the Two Groups

**Figure 4:**
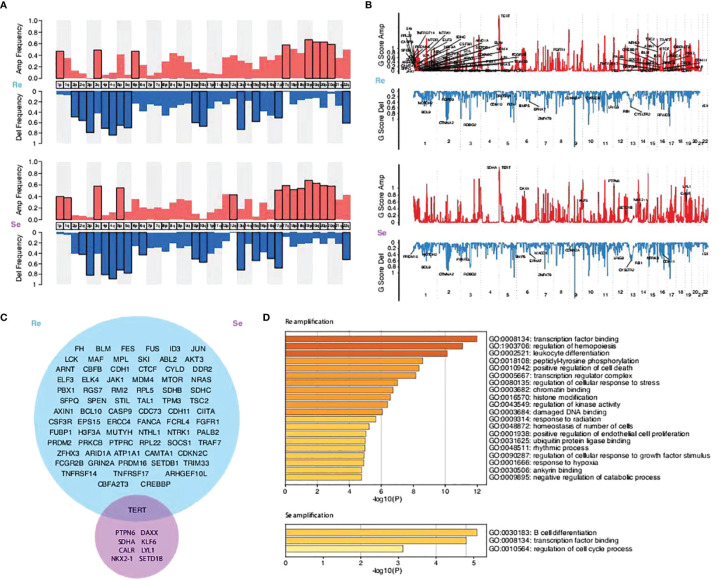
Comparison of the copy number variations between the two groups and their enriched biological functions. **(A)** Amplification and deletion frequency of copy number variations (CNVs) on the chromosome arm level. **(B)** Scores of the significant amplification and deletion regions. **(C)** Venn graphs showing different amplification focal CNV genes between the two groups predicted by the GISTIC method (FDR *q* < 0.1). **(D)** KEGG pathways and GO functions enriched by focal CNV genes that significantly affected PFS time.

The subsequent analysis revealed differences between the two groups. The segmented copy numbers were visualized in a heatmap and the significance of chromosome alterations was determined by GISTIC analysis (**Figures 4A, B**). The differing clones of mutations were prevalent in the two types of specimens with a few similar parts. We found more amplified mutations in the chemorefractory group, which are related to certain functions, such as transcription factor binding, regulation of hemopoiesis, leukocyte differentiation, peptidyl-tyrosine phosphorylation, positive regulation of cell death, transcription regulator complex, regulation of cellular response to stress, chromatin binding, histone modification, regulation of kinase activity, damaged DNA binding, response to radiation, homeostasis of a number of cells, positive regulation of endothelial cell proliferation, ubiquitin protein ligase binding, rhythmic process, regulation of cellular response to growth factor stimulus, response to hypoxia, ankyrin binding, and negative regulation of catabolic process (**Figures 4C, D**). Amplified mutations in the chemosensitive group inhibited the regulation of the cell cycle process, transcription factor binding, and B-cell differentiation. Analysis of deletion mutation also suggested that the detection of the chromosomal-level variation might influence our treatment strategies (**Supplementary Figure 13**).

### Immunotherapy Features Between the Two Groups

**Figure 5:**
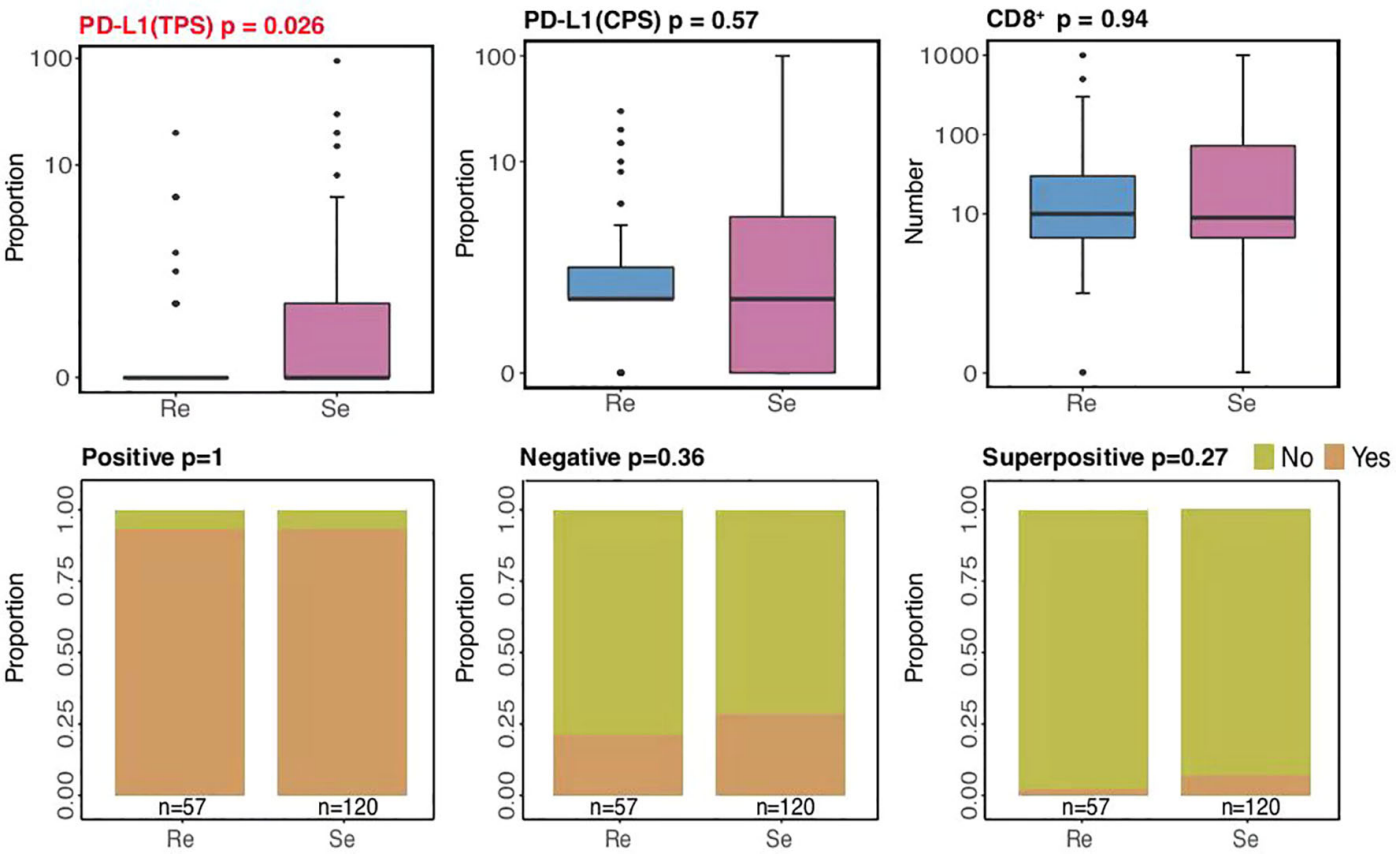
Comparison of immunotherapy-related biomarkers.

Immune checkpoint inhibitors (ICIs) have altered the treatment of SCLC (32–34). Multiple biomarkers, such as PD-L1 and CD8^+^ tumor-infiltrating lymphocytes (TILs), have been identified to help tailor immunotherapy. According to our current study, higher PD-L1 expressions (based on TPS methods) were mostly present among chemosensitive patients (*p* = 0.026; **Figure 5**), while there were no differences between the two groups in terms of PD-L1 expressions (based on CPS methods) and CD8^+^ TILs. Significantly, hyperprogressive disease (HPD), a pattern of progression in which a flare-up of tumor growth occurred during immunotherapy, was similar between the two groups. This suggests that chemosensitive patients might more likely benefit from immunotherapy.

It has been shown that some crucial genetic mutations could influence the efficiency of ICI treatments (35). However, our study suggests that there were no differences in genomic instability between the two groups. Analyses that take more factors into account are needed in our future studies (**Supplementary Figure 14**).

### The Predictive Model of Drug Resistance of SCLC

**Figure 6:**
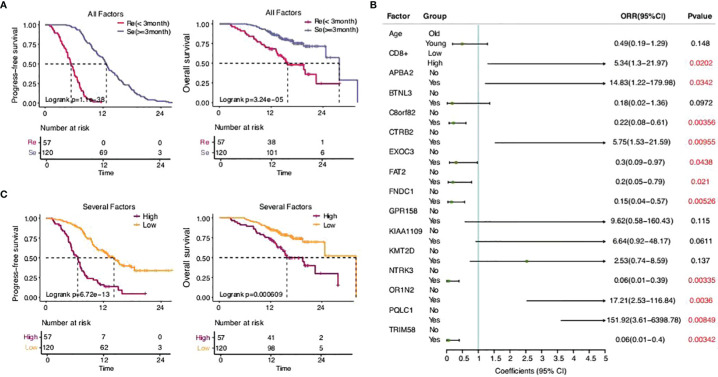
The predictive model of drug resistance of SCLC. **(A)** Statistical analysis of the two groups. **(B)** Sixteen eigenvalues got selected through the stepwise regression process with resistance as the target variable. **(C)** Statistical analysis of the high-risk group and the low-risk group.

There was a significant difference in the survival rates of different resistance levels in SCLC patients (**Figure 6A**). In this study, we attempted to screen optimal subsets of features between the two groups and set up a predictive model of drug resistance of SCLC. The statistical analysis of data from three different angles offered us some clues (**Table 1**; **Supplementary Table 1**). The first part included the clinical characteristics, age, gender, stage, family history, smoking, drinking, metastasis, PD-L1 expressions, and CD8^+^ TILs. The factors with *p*-value ≤0.2 were selected by univariate logistic regression analysis, and age, stage, family history, and CD8^+^ TILs were chosen. Similarly, differences at the molecular level, such as *ABCC1*, *APBA2*, *GPR158*, *KMT2D*, *NTRK3*, *TRIM58*, *FNDC1*, *FAT2*, *OR1N2*, *LRP2*, and *KIAA1109*, were obtained. The same goes for features of chromosome variation that included *C8orf82*, *CTRB2*, *EXOC3*, *PQLC1*, and *BTNL3*.

**Table 1:** Logisic regression models to check the differences in Re and Se groups.

Biomarkers	Group	Odds ratio	95% CI	*p*-value
Age_group	Old			
	Young	0.64	0.34–1.22	0.175
Stage	Extensive stage			
	Limited stage	2.19	1.14–4.2	0.0187
Fam. hist	No			
	Yes	0.61	0.29–1.27	0.187
Gender	Male			
	Female	1.32	0.64–2.7	0.453
Smoking	Yes			
	No	1.43	0.74–2.76	0.289
Drinking	No			
	Yes	0.7	0.37–1.33	0.28
Metastases	No			
	Yes	0.94	0.41–2.17	0.884
CD8_num_group	Low			
	High	2.13	0.91–5	0.0813
PDL1_TPS_prop_group	Low			
	High	1.6	0.6–4.25	0.347
*ABCC1*	No			
	Yes	22,454,206.5	0–Inf	0.988
*APBA2*	No			
	Yes	6.8	0.87–53.35	0.068
*BTNL3*	No			
	Yes	0.29	0.1–0.81	0.0182
*C8orf82*	No			
	Yes	0.46	0.23–0.89	0.0222
*CTRB2*	No			
	Yes	3.31	1.21–9.09	0.0199
*EXOC3*	No			
	Yes	0.31	0.14–0.7	0.00504
*FAT2*	No			
	Yes	0.38	0.16–0.92	0.0321
*FNDC1*	No			
	Yes	0.34	0.14–0.82	0.0162
*GPR158*	No			
	Yes	7.4	0.95–57.71	0.0563
*KIAA1109*	No			
	Yes	3.39	0.96–11.96	0.0581
*KMT2D*	No			
	Yes	2.63	1.13–6.1	0.0249
*NTRK3*	No			
	Yes	0.27	0.08–0.85	0.0262
*OR1N2*	No			
	Yes	3.82	1.09–13.38	0.0363
*PQLC1*	No			
	Yes	9.24	1.2–71.28	0.0329
*TRIM58*	No			
	Yes	0.2	0.07–0.63	0.0057
*LRP2*	No			
	Yes	2.63	1.13–6.1	0.0249

There were 20 eigenvalues in total as discussed previously. Step function was used to determine multivariate logistic regression through a stepwise regression process with resistance as the target variable. Eventually, 16 eigenvalues were selected (**Figures 6B, C**).

According to the model determined by logistic regression, each sample was endowed with a predictive probability value (PV). Then, the samples were divided into a high-risk group (>0.55) and a low-risk group (≤0.55), and the survival analysis showed obvious differences between the groups. This suggests that the predictive model, to a certain extent, could predict the drug resistance of SCLC.

## Discussion

The treatment of lung cancer has achieved remarkable progress in the past two decades and has improved the outcomes for many patients. The in-depth study of driver genes has realized the individualized treatment of patients with NSCLC (36–39) and significantly improved the survival time. However, this advantage did not benefit patients with SCLC. At present, SCLC is divided into different subtypes (40), which had no substantial significance in clinical therapeutic decision-making. ICIs have also improved the prognosis of SCLC to a certain extent, but the treatment options are mainly refined to radiotherapy and chemotherapy. Chemotherapy resistance is one of the main reasons for the poor prognosis of SCLC. From a clinical perspective, SCLC is generally divided into chemorefractory and chemosensitive according to PFS time. In this case, there is neither an effective means to evaluate the potential benefits of patients before treatment nor a molecular mechanism to further explore effective treatment methods.

Genetic mutations are widely present in two different types of patients, which is the same as previously reported. Our SNV and CNV analyses showed a significant difference between the two groups. Further functional analysis showed that genomic instability in cancer cells may lead to the tumor’s rapid growth, tendency to metastasize, immune escape, and resistance to chemotherapy. Interestingly, the mutation status of *LRP2* related to encoding for low-density lipoprotein-related protein 2 or megalin affected the OS time, while other gene mutations only affected the PFS time. The particularity of *LRP2* deserves further study. Targeted screening and evaluation by WES providing the reference criterion for individualized treatment and drug development are imperatively needed.

There have been some particular findings in chromosomal-level genomic alterations. The amplification mutation of the chemorefractory group increased significantly and was significantly related to tumor proliferation, metastasis, and immune escape. This shows that genomic heterogeneity is the main reason for the different biological behaviors. Similarly, the difference in deletion mutation between the two groups was more pronounced in cell proliferation in the chemorefractory group (41). Interestingly, it has been indicated by some studies that inactivating the *ERK1/2* signaling pathway would suppress cisplatin resistance in non-small cell lung cancer (42). It deeply supported the correlation of drug resistance and provided a basis for drug research and development.

Immunotherapy, especially with ICIs targeting PD-L1, has durably changed the treatment for SCLC. According to our current study, higher PD-L1 expressions (based on TPS methods) were mostly present among the chemosensitive patients, with HPD being similar between the two groups. Conventional markers, including PD-L1 and CD8^+^ TILs, may not be enough to serve as clinical references (43–46). We observed that mutations in our experimental samples were related to functions and immune responses, such as leukocyte differentiation ratio, regulation of cellular response to stress, and B-cell differentiation, which warrants the prediction of immunotherapy benefits by immune-related markers and genetic background incorporation. Altogether, this suggested that chemosensitive patients might be the most appropriate subgroup for immunotherapy. However, a broader analysis of predictive biomarkers should be carried out in the future to verify our inferences.

There have been several studies establishing the corresponding classifier for clinical outcomes based on a single CTC in patients with SCLC (4, 5). We screened a wider range of features that covered clinical characteristics, molecular level, and chromosome variation. The survival analysis showed an obvious advantage in the low-risk group (*p* = 6.72e−13) suggesting that our model has significant potential as a predictive and prognostic method. However, it needs to be proven by further experiments using larger samples.

Overall, our study has expanded our knowledge regarding SCLC based on a total of 177 patients with SCLC, the largest Chinese SCLC cohort study to date. Our findings revealed the difference between the two groups, the genomic characteristics, and the resistance mechanism of Chinese SCLC patients, thereby laying the groundwork for improved SCLC management *via* personalized medicine development. The model established by logistic regression divided patients into high-risk and low-risk groups, so as to establish a convenient approach for clinical disease differentiation. This study provides a reference basis to translate knowledge into practice, such as formulating personalized treatment plans, which may benefit Chinese patients with SCLC.

## Data Availability Statement

The datasets presented in this study can be found in online repositories. The names of the repository/repositories and accession number(s) can be found below: Genome Sequence Archive in Data Center of Beijing Institute of Genomics under the accession number subHRA001430.

## Author Contributions

HW and JG: conceptualization, writing—review and editing, supervision, and validation. NT: writing—original draft, visualization, methodology, investigation, and formal analysis. ZL, XH, and CZ: writing—methodology and formal analysis. All authors contributed to the article and approved the submitted version.

## Funding

This study was supported jointly by special funds for Taishan Scholars Project (grant no. tsqn201812149).

## Conflict of Interest

ZL was employed by Berry Oncology Corporation.

The remaining authors declare that the research was conducted in the absence of any commercial or financial relationships that could be construed as a potential conflict of interest.

## Publisher’s Note

All claims expressed in this article are solely those of the authors and do not necessarily represent those of their affiliated organizations, or those of the publisher, the editors and the reviewers. Any product that may be evaluated in this article, or claim that may be made by its manufacturer, is not guaranteed or endorsed by the publisher.

## Supplementary Material

Supplementary Figure 1 Comparison of clinical features between the two group.

Supplementary Figure 2 Comparison of four highly confident mutational signatures (S1, S2, S3, and S4) derived in the SCH cohort with those in the COSMIC dataset using Cosine similarity analysis.

Supplementary Figure 3 PFS of OR4C6 different mutation status.PFS, progression-free survival.

Supplementary Figure 4 PFS of PTPN13different mutation status.

Supplementary Figure 5 OS of *LRP2*different mutation status.OS, overall survival.

Supplementary Figure 6 TheVAF analysis of the two group.VAF, variant allele fraction.

Supplementary Figure 7 PFS of CTDP1different mutation status.

Supplementary Figure 8 PFS of KLF5different mutation status.

Supplementary Figure 9 PFS of NFATC1different mutation status.

Supplementary Figure 10 PFS of SYTL1different mutation status.

Supplementary Figure 11 PFS of TTLL10different mutation status.

Supplementary Figure 12 OS of PQLC1different mutation status.

Supplementary Figure 13 Comparison of copy number variations between the two group and their enriched biological functions. **(A)** Venn graphs showing different deletionfocal CNV genes between the two group predicted by the GISTIC method (FDR q < 0.1). **(B)** KEGG pathways and GO functions enriched by focal CNV genes that significantly affected PFS time.

Supplementary Figure 14 Comparison of genomic instability between two groups.

Supplementary Table 1 Eigenvalues of the two groups in total.
