# Supplementary figures and images for: Whole-Exome Sequencing Uncovers Specific Genetic Variation Difference Based on Different Modes of Drug Resistance in Small Cell Lung Cancer
Source: Front Oncol. 2022 Jun 30;12:891938. doi: 10.3389/fonc.2022.891938 (PMC9280676; doi:10.3389/fonc.2022.891938)

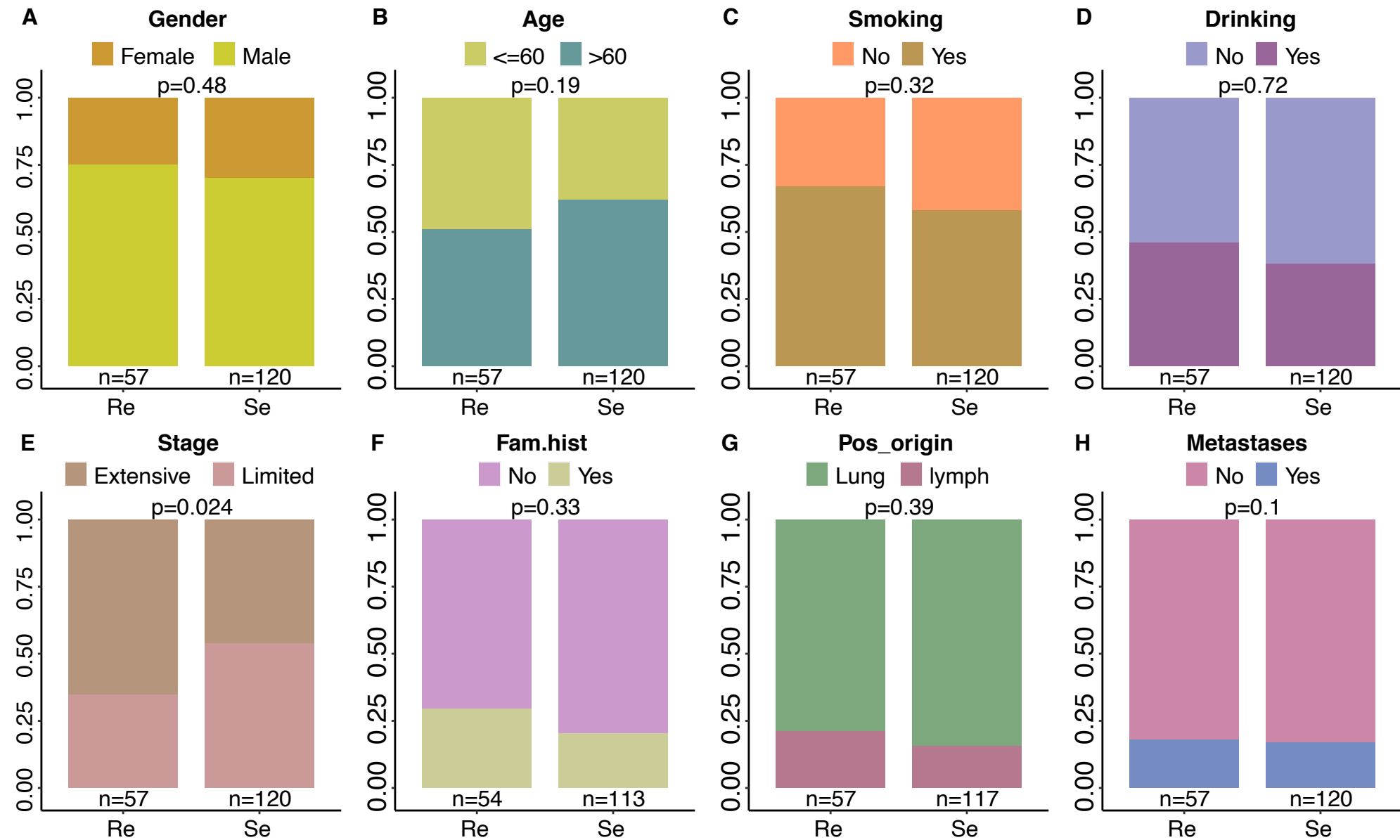

Supplement: Supplementary Figure 1 — Comparison of clinical features between the two group. [file DataSheet_1.pdf]

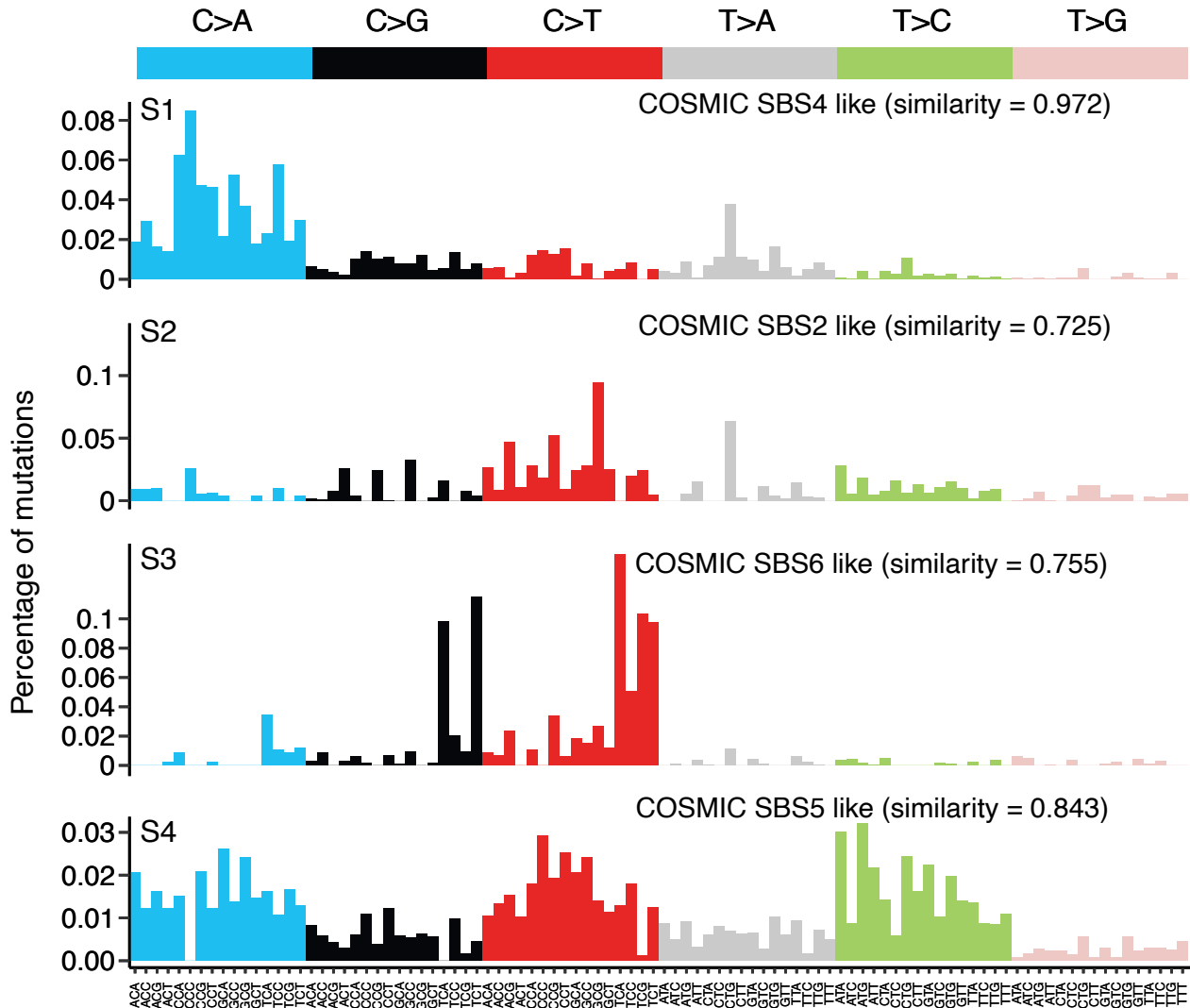

Supplement: Supplementary Figure 2 — Comparison of four highly confident mutational signatures (S1, S2, S3, and S4) derived in the SCH cohort with those in the COSMIC dataset using Cosine similarity analysis. [file DataSheet_2.pdf]

# OR4C6

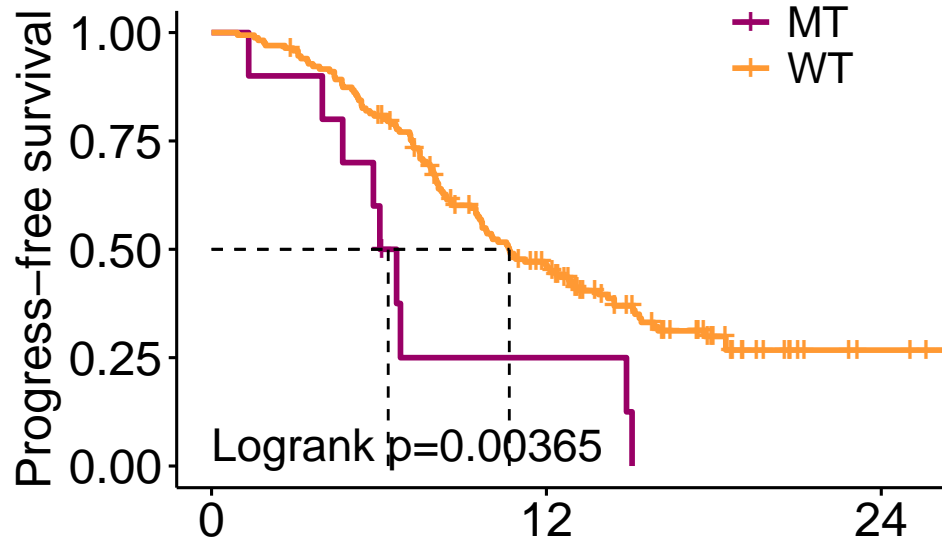

Number at risk

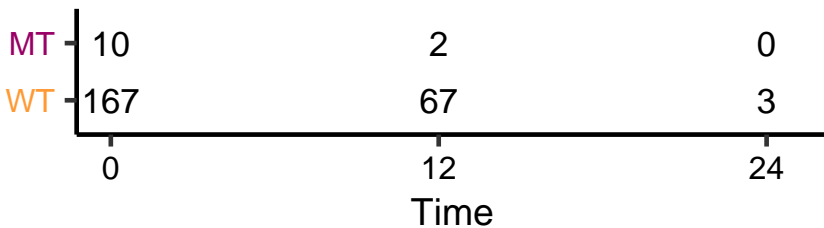

Supplement: Supplementary Figure 3 — PFS of OR4C6 different mutation status.PFS, progression-free survival. [file DataSheet_3.pdf]

# PTPN13

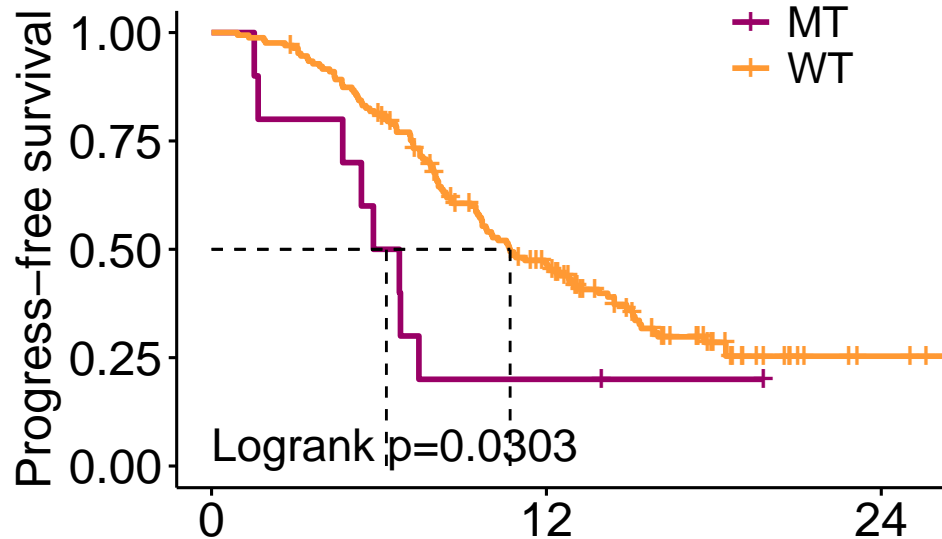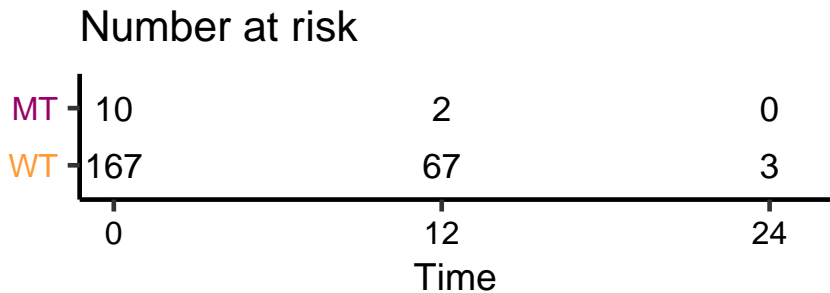

Supplement: Supplementary Figure 4 — PFS of PTPN13different mutation status. [file DataSheet_4.pdf]

# LRP2

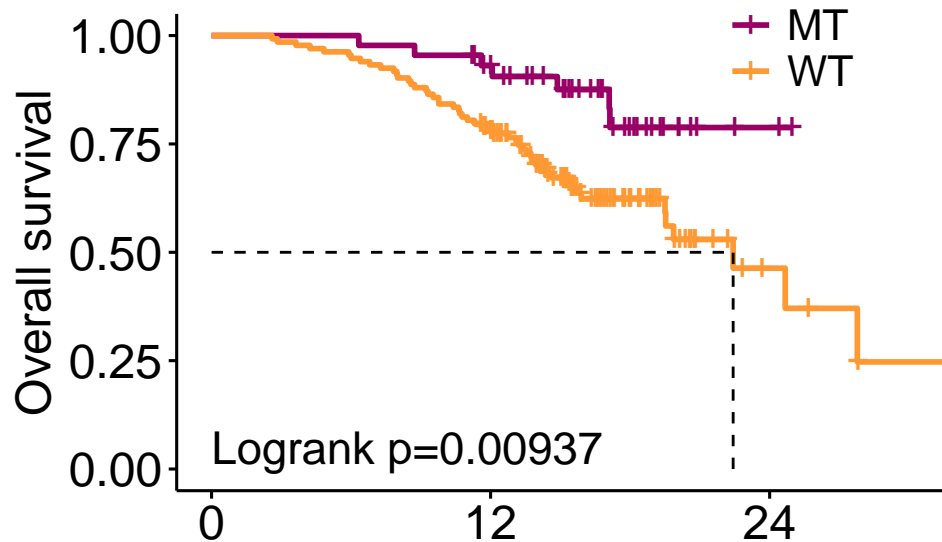

Number at risk

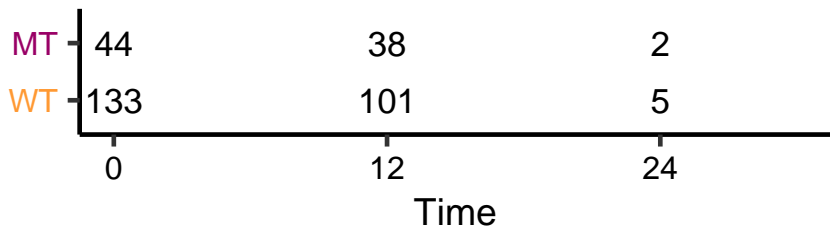

Supplement: Supplementary Figure 5 — OS of LRP2different mutation status.OS, overall survival. [file DataSheet_5.pdf]

**A****ALL**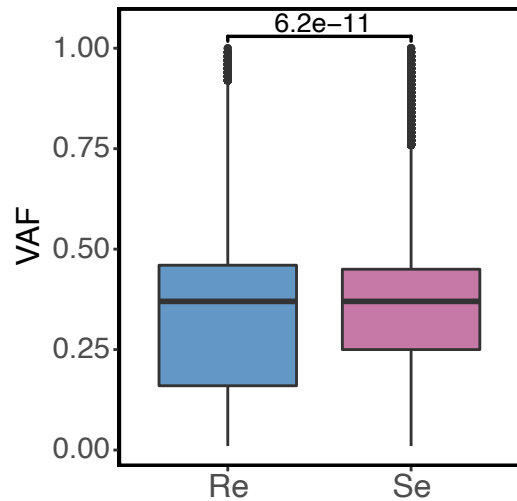**Clonal**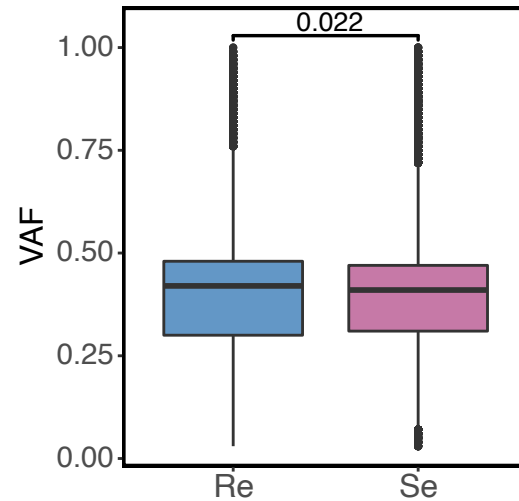**B****TMB**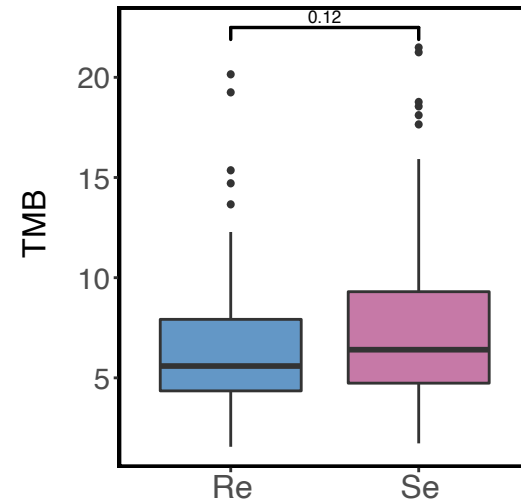**C****Math**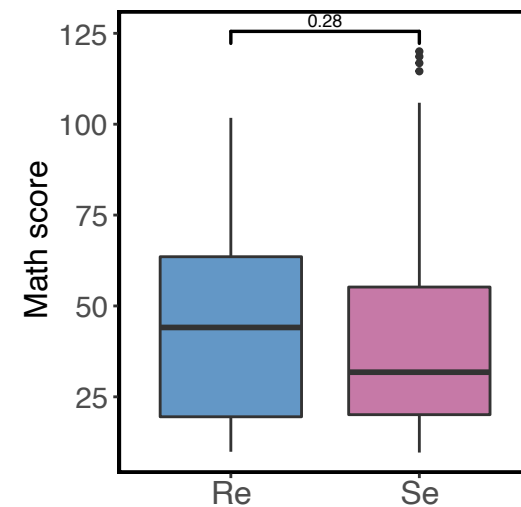**Driver**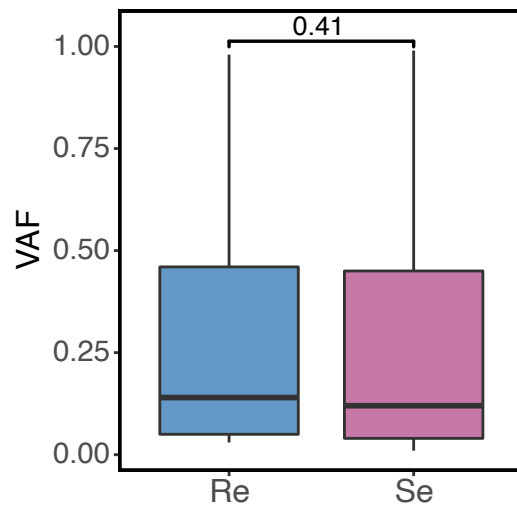**LOH**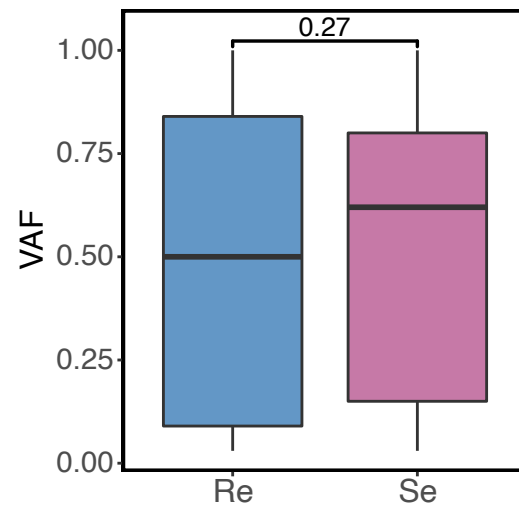

Supplement: Supplementary Figure 6 — TheVAF analysis of the two group.VAF, variant allele fraction. [file DataSheet_6.pdf]

## CTDP1

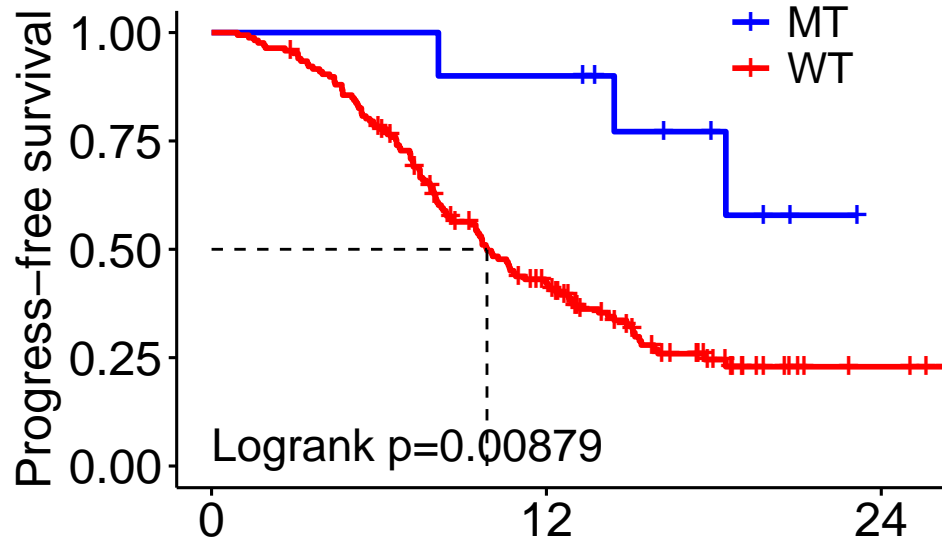

Number at risk

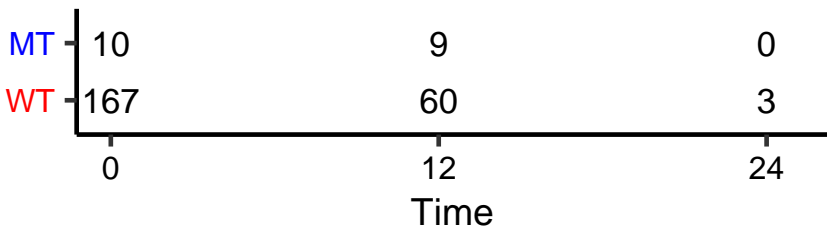

Supplement: Supplementary Figure 7 — PFS of CTDP1different mutation status. [file DataSheet_7.pdf]

# KLF5

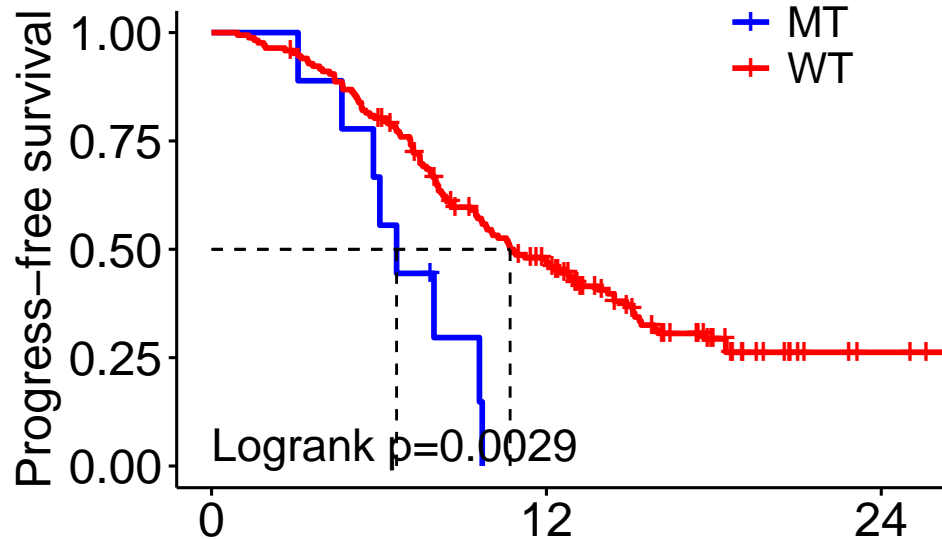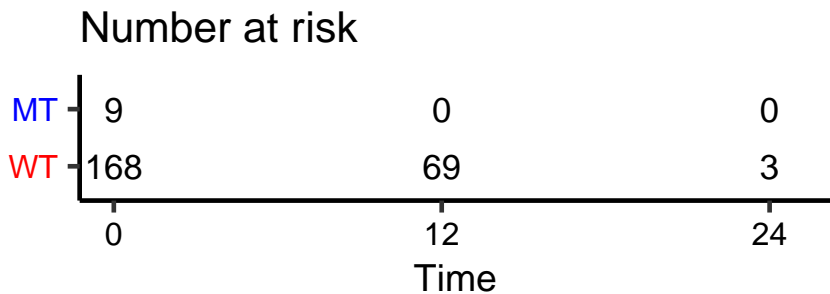

Supplement: Supplementary Figure 8 — PFS of KLF5different mutation status. [file DataSheet_8.pdf]

# NFATC1

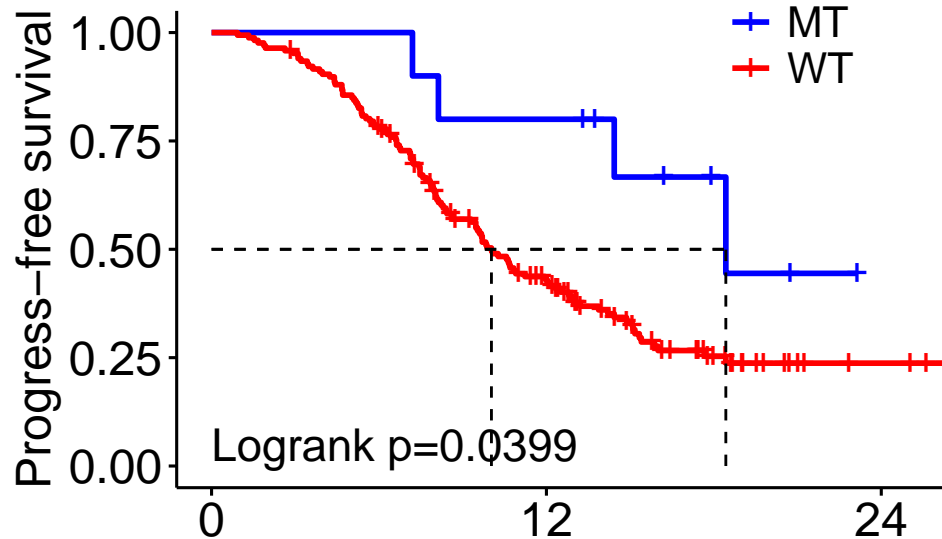

Number at risk

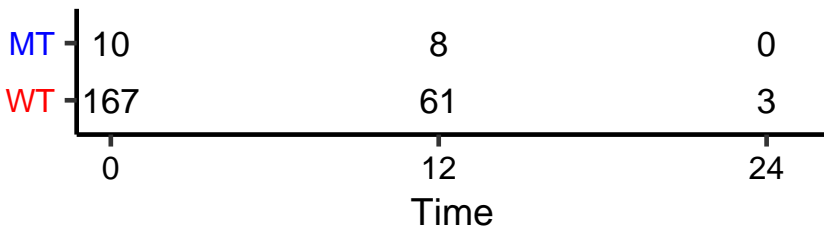

Supplement: Supplementary Figure 9 — PFS of NFATC1different mutation status. [file DataSheet_9.pdf]

# SYTL1

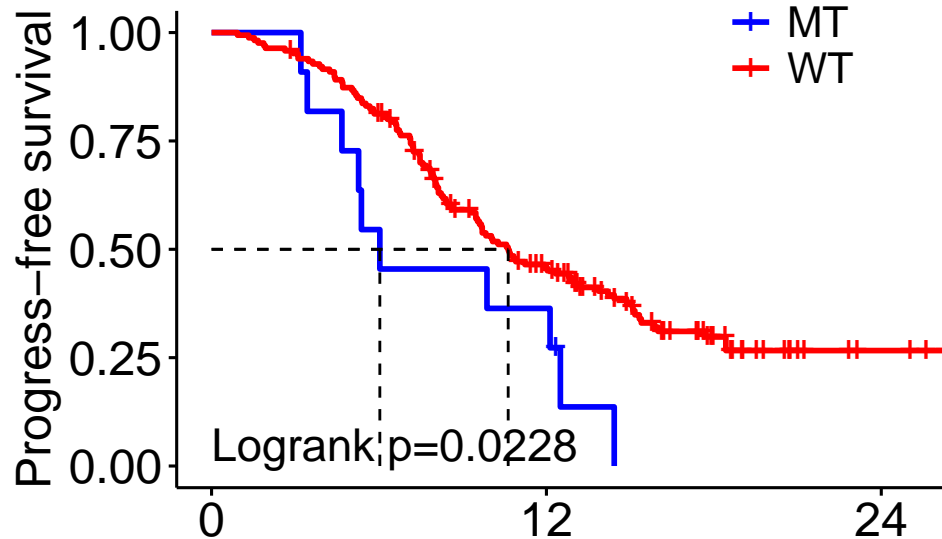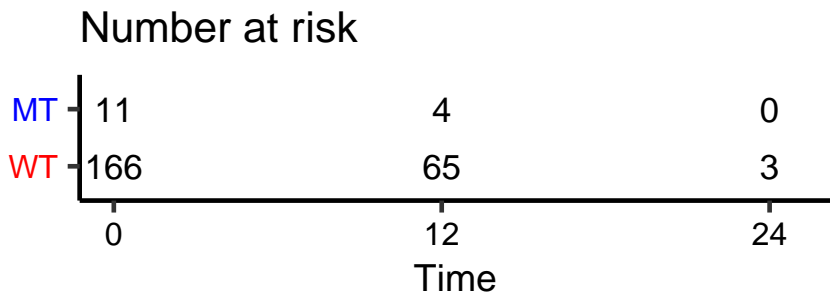

Supplement: Supplementary Figure 10 — PFS of SYTL1different mutation status. [file DataSheet_10.pdf]

# TTLL10

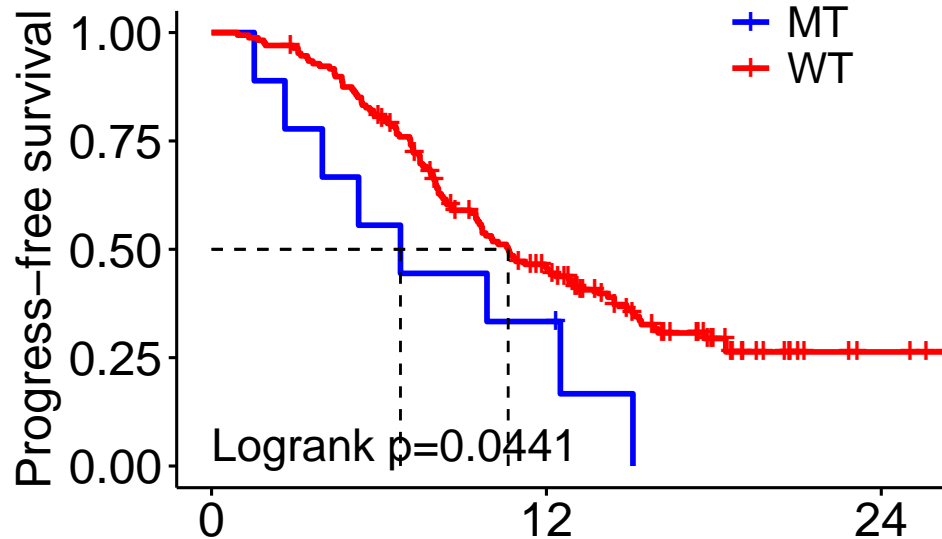

Number at risk

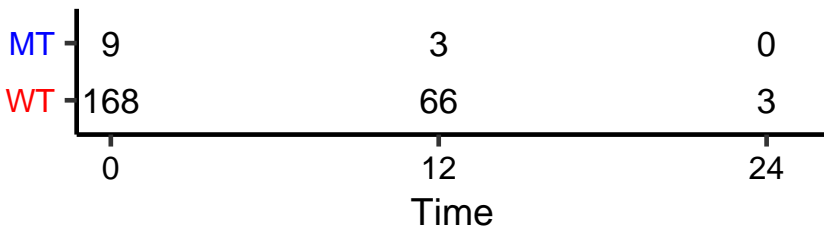

Supplement: Supplementary Figure 11 — PFS of TTLL10different mutation status. [file DataSheet_11.pdf]

# PQLC1

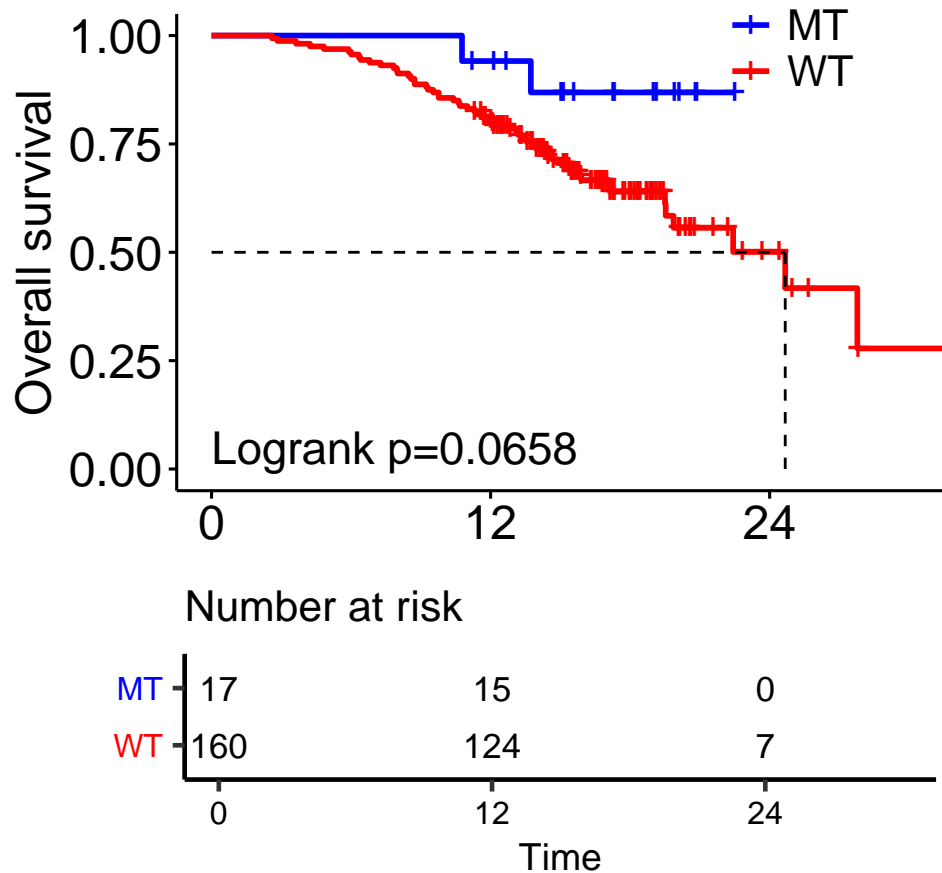

Supplement: Supplementary Figure 12 — OS of PQLC1different mutation status. [file DataSheet_12.pdf]

**LOH  $p = 0.16$**

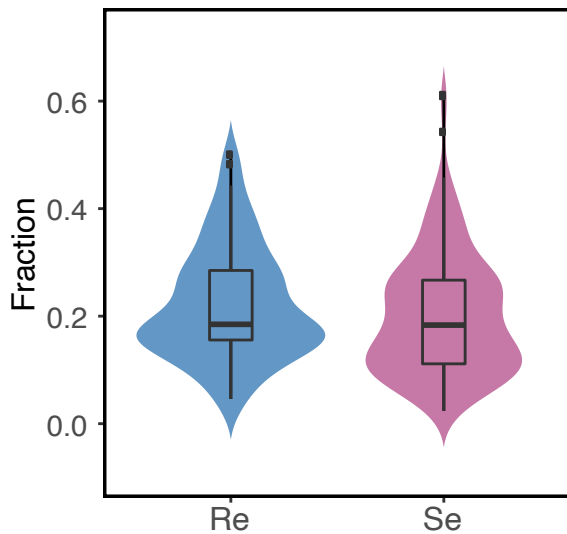

**FGA  $p = 0.34$**

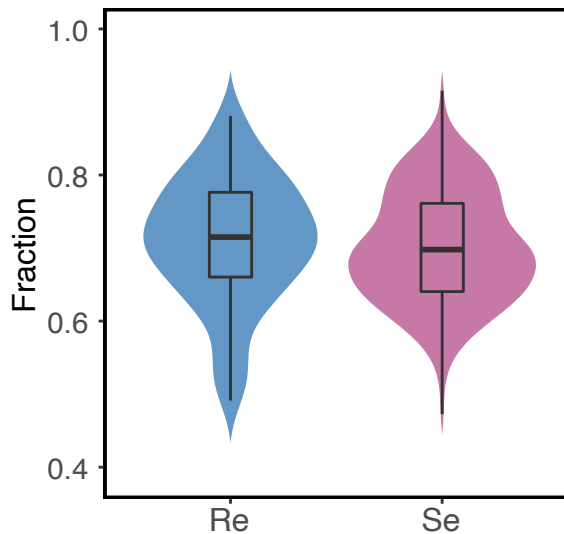

**Ploidy  $p = 0.36$**

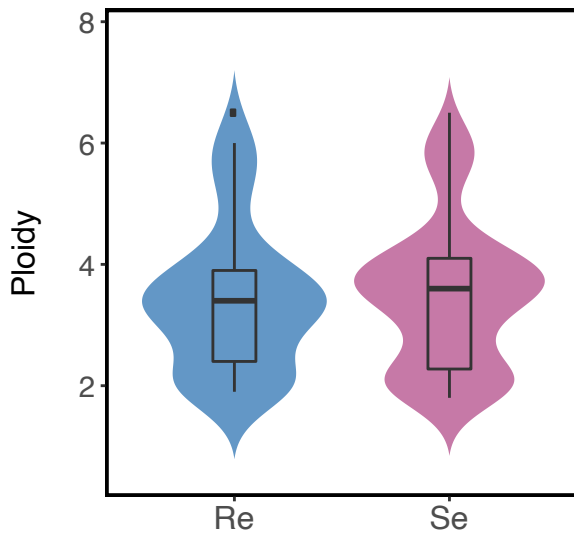

**WGD  $p = 1$**

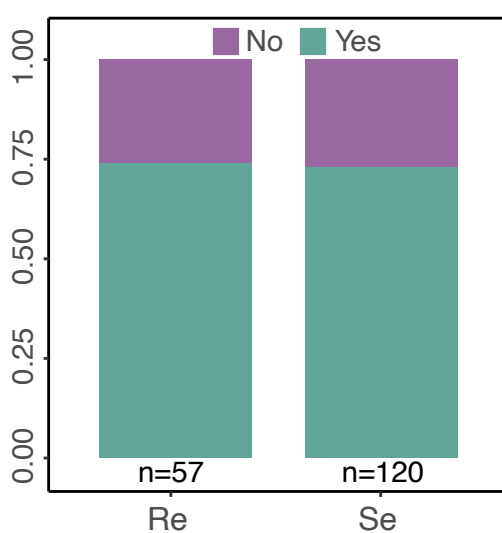

Supplement: Supplementary Figure 14 — Comparison of genomic instability between two groups. [file DataSheet_14.pdf]
