# Supplementary material for: Whole-Exome Sequencing Uncovers Specific Genetic Variation Difference Based on Different Modes of Drug Resistance in Small Cell Lung Cancer
Source: Front Oncol. 2022 Jun 30;12:891938. doi: 10.3389/fonc.2022.891938 (PMC9280676; doi:10.3389/fonc.2022.891938)
Supplement: Supplementary Figure 13 — Comparison of copy number variations between the two group and their enriched biological functions. (A) Venn graphs showing different deletionfocal CNV genes between the two group predicted by the GISTIC method (FDR q < 0.1). (B) KEGG pathways and GO functions enriched by focal CNV genes that significantly affected PFS time. [file DataSheet_13.pdf]

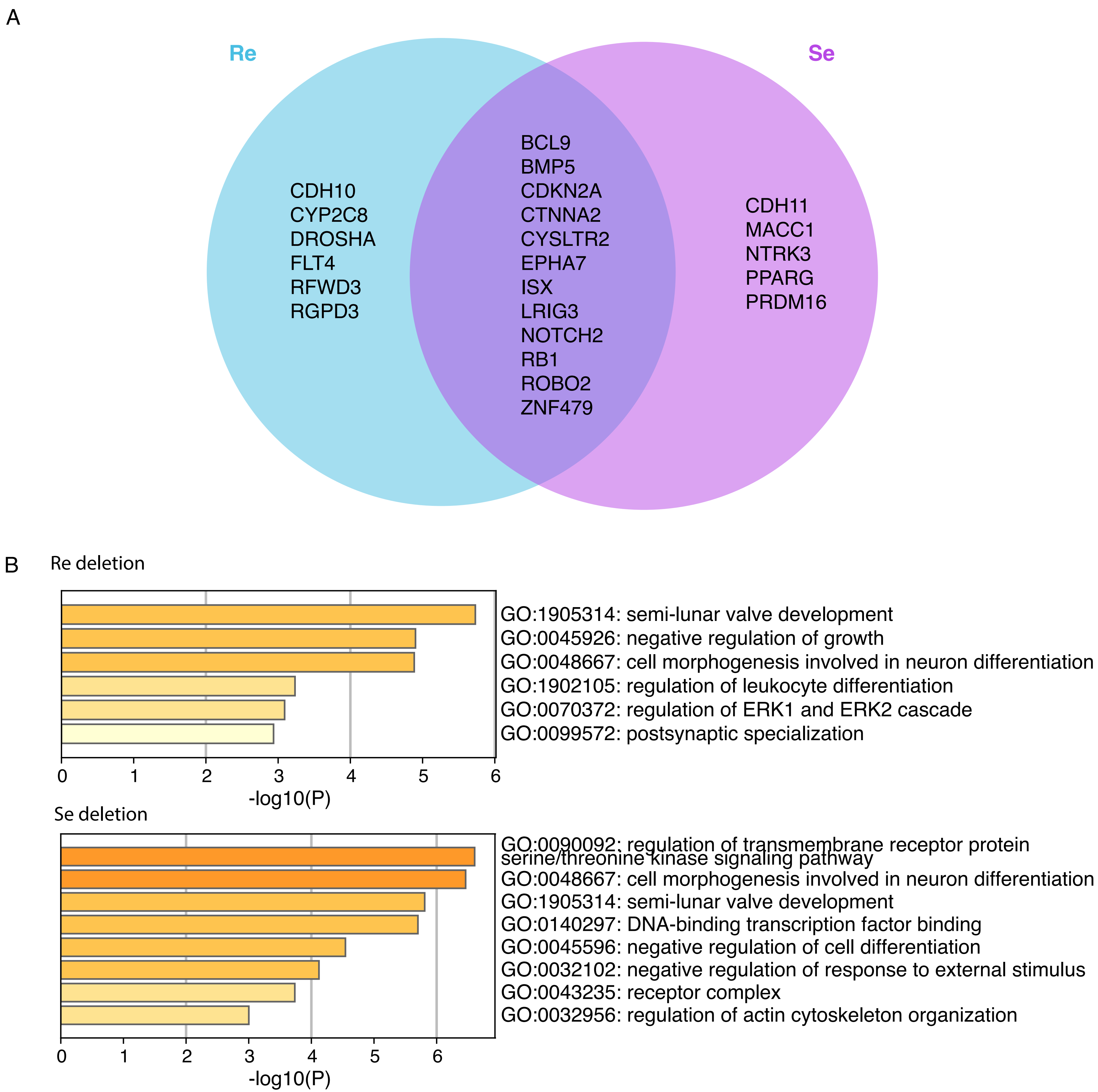
A
Re
Se
CDH10
CYP2C8
DROSHA
FLT4
RFWD3
RGPD3
BCL9
BMP5
CDKN2A
CTNNA2
CYSLTR2
EPHA7
ISX
LRIG3
NOTCH2
RB1
ROBO2
ZNF479
CDH11
MACC1
NTRK3
PPARG
PRDM16
B
Re deletion
GO:1905314: semi-lunar valve development
GO:0045926: negative regulation of growth
GO:0048667: cell morphogenesis involved in neuron differentiation
GO:1902105: regulation of leukocyte differentiation
GO:0070372: regulation of ERK1 and ERK2 cascade
GO:0099572: postsynaptic specialization
0
1
2
3
4
5
6
-log10(P)
Se deletion
GO:0090092: regulation of transmembrane receptor protein serine/threonine kinase signaling pathway
GO:0048667: cell morphogenesis involved in neuron differentiation
GO:1905314: semi-lunar valve development
GO:0140297: DNA-binding transcription factor binding
GO:0045596: negative regulation of cell differentiation
GO:0032102: negative regulation of response to external stimulus
GO:0043235: receptor complex
GO:0032956: regulation of actin cytoskeleton organization
0
1
2
3
4
5
6
-log10(P)
